# Trans-spinal magnetic stimulation upregulates microglial SOCS3 to attenuate neuroinflammation in chronic constriction injury–induced neuropathic pain

**DOI:** 10.4103/NRR.NRR-D-24-00912

**Published:** 2025-04-29

**Authors:** Qi Wu, Xingjun Xu, Chenyuan Zhai, Jili Cai, Zun Wang, Lu Fang, Yu Wang, Yilun Qian, Manyu Dong, Liang Hu, Tong Wang, Ying Shen, Wentao Liu

**Affiliations:** 1 Department of Rehabilitation, Hengyang Medical School, The First Affiliated Hospital, University of South China, Hengyang, Hunan Province, China; 2 Rehabilitation Medicine Center, The First Affiliated Hospital of Nanjing Medical University (Jiangsu Province Hospital), Nanjing, Jiangsu Province, China; 3 Department of Rehabilitation, Southwest Hospital, Third Military Medical University (Army Medical University), Chongqing, China; 4 Department of Rehabilitation Medicine, The Affiliated Suzhou Hospital of Nanjing Medical University, Suzhou Municipal Hospital, Gusu School, Nanjing Medical University, Suzhou, Jiangsu Province, China; 5 Jiangsu Key Laboratory of Neurodegeneration, Department of Pharmacology, Nanjing Medical University, Nanjing, Jiangsu Province, China

**Keywords:** CaMKKβ/AMPK/SOCS3 signaling, chronic constriction injury, mechanical pain sensitivity, microglia, neuropathic pain, neuroinflammation, nuclear factor-κB p65, repetitive trans-spinal magnetic stimulation, STAT3

## Abstract

Current treatments for neuropathic pain are suboptimal, necessitating the search for more effective therapeutics. Our previous study showed that inhibition of neuroinflammation in the spinal cord induced analgesic effects, and focal repetitive trans-spinal magnetic stimulation showed an anti-neuroinflammatory effect in spinal cord injury rat models. Here, we speculated that repetitive trans-spinal magnetic stimulation might induce an anti-inflammatory effect to alleviate neuropathic pain by upregulating calmodulin-dependent protein kinase kinase beta (CaMKKβ)/adenosine 5′-monophosphate-activated protein kinase (AMPK)/suppressor of cytokine signaling-3 (SOCS3) signaling in microglia. Experiments have found that non-invasive focal repetitive trans-spinal magnetic stimulation effectively alleviates mechanical allodynia and spinal neuroinflammation in rats with neuropathic pain induced by chronic sciatic nerve ligation. Further research found that repetitive trans-spinal magnetic stimulation upregulated the expression of SOCS3 in spinal microglia, which subsequently inhibited the phosphorylation of p38 mitogen-activated protein kinase and signal transducer and activator of transcription 3 and nuclear factor-kappa B p65 nuclear translocation in rats with neuropathic pain, thereby suppressing neuroinflammation. The upregulation of SOCS3 by repetitive trans-spinal magnetic stimulation may be achieved through the activation of the CaMKKβ/AMPK signaling pathway in microglia. The results suggested that focal repetitive trans-spinal magnetic stimulation inhibits spinal neuroinflammation and alleviates neuropathic pain by activating the CaMKKβ/AMPK/SOCS3 signaling pathway in spinal microglia. This mechanism provides an effective noninvasive treatment for neuropathic pain caused by peripheral nerve injury.

## Graphical abstract

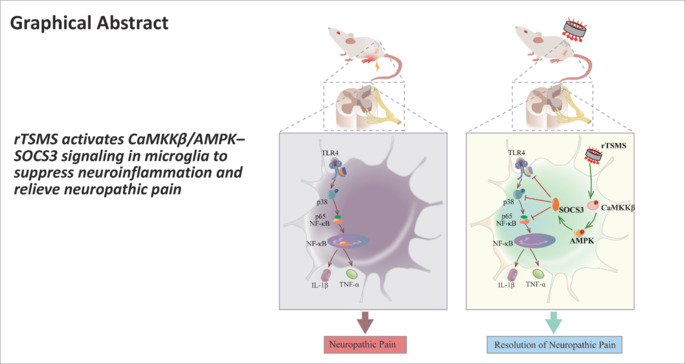

## Introduction

Neuropathic pain (NP), a devastating form of chronic pain, imposes considerable social and economic burdens on patients and society worldwide (Bouhassira, 2019). The management of NP is challenging as currently available drugs have poor efficacy and are associated with severe adverse effects and addiction. It is generally believed that neuroinflammation in the dorsal horn, involved in immune cell recruitment and the release of mediators, plays a central role in the chronification and central sensitization of NP (Sommer et al., 2018). In the last decade, preclinical studies of NP have explored neuroinflammation and glial activity in the dorsal horn, finding that NP is attenuated by neuroinflammation inhibition and glial activation (Hu et al., 2020; Yu et al., 2020a; Sun et al., 2021). Systemic inhibition of neuroinflammation and glial activation may have serious side effects, and there is currently no effective means of targeted drug delivery to the dorsal horn for clinical treatment of NP. Therefore, a novel noninvasive targeted therapy to suppress neuroinflammation in the dorsal horn is a promising therapeutic strategy for NP.

Neuroinflammation is a major cause of chronic pain (Wu et al., 2025). Neuroinflammation in the spinal cord drives chronic pain via neuron–glial interactions (Yi et al., 2021). Peripheral nerve injury promotes de novo expression of colony-stimulating factor 1, inducing microglial proliferation and activation in the dorsal horn of the spinal cord. The diffusion of neuroinflammation in the central nervous system is underpinned by microglial activation (Guan et al., 2016). The activated microglia release the majority of proinflammatory cytokines, such as interleukin-1 beta (IL-1β), interleukin (IL)-6, and tumor necrosis factor-alpha (TNF-α), which further activate the adjacent glia to induce neuroinflammation diffusion and contribute to chronic pain states (Chen et al., 2018). This finding suggests that targeting neuroinflammation may be a therapeutic strategy for chronic pain.

Electromagnetic biology is an emerging medical field with broad application prospects. Transcranial magnetic stimulation is a safe and noninvasive brain stimulation technique based on electromagnetic induction via a wire coil generating a magnetic field that passes through the scalp. Repetitive transcranial magnetic stimulation (rTMS) has been used clinically to treat neuropsychiatric diseases (Lefaucheur et al., 2020) and exhibits anti-inflammatory effects (Luo et al., 2022b). So far, few studies have applied focal and noninvasive repetitive trans-spinal magnetic stimulation (rTSMS) for treating NP, and the underlying cellular and molecular mechanisms are yet to be elucidated. Our previous study showed that focal rTSMS is a noninvasive spinal cord injury (SCI) treatment that reduces neuroinflammation (Zhai et al., 2024). Therefore, we speculate that rTSMS might be a promising analgesic strategy for chronic pain. However, whether rTSMS is associated with neuroinflammation regulation in chronic pain treatment warrants further investigation.

Suppressor of cytokine signaling 3 (SOCS3), a “powerful brake” in cytokine signaling pathways, negatively regulates the inflammatory response by inhibiting Toll-like receptor 4 (TLR4), IL-1 receptor, and IL-6 receptor signaling (Baker et al., 2009). Our previous study showed that SOCS3 upregulation induces inflammatory tolerance and relieves incision-induced mechanical allodynia (Qian et al., 2023). Studies have also demonstrated that rTMS inhibits TLR4 and IL-1 signaling in different disease models (Zhao et al., 2019; Luo et al., 2022b). Thus, rTSMS might inhibit neuroinflammation for analgesic purposes by upregulating SOCS3. Therefore, this study aims to investigate whether rTSMS can provide relief from NP and elucidate the underlying mechanisms of its analgesic effects.

## Methods

### Ethics statement

All experiments were designed and reported according to the Animal Research: Reporting of *In Vivo* Experiments guidelines (Percie du Sert et al., 2020). All animal experiments were approved by Nanjing Medical University Animal Care and Use Committee (approval No. IACUC-2103061 [16 March 2021]) and were designed to minimize suffering of the animals.

### Animals and NP model

Adult female Sprague–Dawley rats (aged 8 weeks, weight of 180–220 g; *n* = 104) were obtained from the Experimental Animal Center of Nanjing Medical University (Nanjing, China; animal license No. SYXK (Su) 2020-0023). The rats (three per cage) were raised under pathogen-free conditions in cages with soft bedding under controlled temperature (22 ± 2°C), a 12-hour light/dark cycle (lights on at 8:00 a.m.) and 55% ± 5% relative humidity. They were allowed to acclimate to these conditions for at least 7 days before experimentation. Behavioral testing was performed during the light cycle (between 8:30 a.m. and 12:00 p.m.) before the first daily focal rTSMS intervention. The rats were randomly divided into Sham, chronic constriction injury (CCI), CCI + rTSMS, Sham + Vehicle, CCI + Vehicle, CCI + rTSMS + SOCS3 small interfering RNA (siRNA), CCI + rTSMS + Ctr siRNA, Sham + Vehicle, CCI +Vehicle, CCI + rTSMS + Vehicle, and CCI + rTSMS + Compound C groups (the sample size was determined on the basis of prior experience, in line with the principle of scientifically justified use). For behavioral testing, molecular testing, and immunofluorescence, each group comprised eight, five, and three rats, respectively.

CCI of the sciatic nerve was conducted bilaterally, and the model was constructed as described previously (Bennett and Xie, 1988). All surgical operations were carried out under anesthesia induced by pentobarbital (50 mg/kg, intraperitoneal). Next, approximately 7 mm of the sciatic nerve was separated and loosely tied with four ligatures (4-0 chronic gut sutures), spaced 1 mm apart, at the mid-thigh level. After surgery, the skin layers and muscles were sutured, and the surgical area was sterilized with 75% ethyl alcohol. In the Sham and Sham + Vehicle groups, the sciatic nerve was exposed but not ligated.

### Focal repetitive trans-spinal magnetic stimulation

A circular coil (inner diameter, 25 mm; outer diameter, 50 mm) connected to a MagStim Rapid stimulator (Yiruide Medical Equipment Co., Ltd., Wuhan, China) was used to administer the focal rTSMS treatment. The center of the coil was positioned at the lumbosacral enlargement of the spinal cord, specifically at the level of the L1 vertebra. The exact position of the coil was defined when the bilateral hindlimbs and tail involuntarily twitched during coil activation. The movement of animals and position of the coil were controlled manually, with gloves covering the animals’ heads during stimulation.

*In vivo*, the rats were administered a 10-minute focal rTSMS treatment twice a day while they were awake, in the morning and afternoon, and the interval between the two rTSMS interventions was more than 6 hours. Each treatment stimulation session consisted of 20 Hz stimulation with a 5-second continuous pulse train duration and intertrain intervals of 10 seconds. In total, there were 40 trains with 4000 total daily pulses delivered at an intensity of 0.152 T. The intensity of the magnetic stimulation was set to the minimum level that caused involuntary twitches in both the hind legs and tail of all rats when the coil was activated. Sham treatments were administered by holding the coil 10 cm above the vertebrae, ensuring the animals experienced the clicking sound of the coil but received no actual stimulation to the spinal cord.

*In vitro*, the trains of repetitive magnetic stimulation (rMS) were administered as 1000 pulses comprising 10 continuous trains of 100 pulses delivered at 10 Hz (10 seconds/train, 3-second inter-train interval). The intensity was 0.038 T, which was 1% of the rMS device’s maximum power. The center of the coil was placed over the center of the culture plate.

### Behavioral test

Behavioral tests were carried out in a blinded manner. The rats were acclimatized to the testing environment, daily for at least 7 days, before baseline testing and were allowed 30 minutes to acclimatize in the testing apparatus before each test. The mechanical withdrawal threshold was recorded by the Von Frey hairs test (Woodland Hills, Los Angeles, CA, USA) to evaluate mechanical sensitivity (Chaplan et al., 1994). The right plantar surface of each hind paw was stimulated with a series of Von Frey hairs with logarithmically increasing stiffness, applied perpendicular to the plantar surface. Each rat was tested three times, and the average threshold was calculated. Behavioral tests were carried out between 8:30 a.m. and 12:00 p.m. before the first focal rTSMS intervention of the day to avoid any impact of potential circadian variations.

### Intrathecal injection

Intrathecal injections of compound C, an adenosine 5′-monophosphate-activated protein kinase (AMPK) inhibitor (10 µg/10 µL; MedChemExpress, Newark, NJ, USA, Cat# HY-13418A), and SOCS3 siRNA (500 pmol/10 μL; Santa Cruz Biotechnology, Santa Cruz, CA, USA, Cat# SC-270156) were performed as described previously by Mestre et al. (1994). The rats were placed in a prone position, supported by a 50-mL conical tube, to identify the intervertebral space between the L5 and L6 vertebrae. A Hamilton syringe with a 30-gauge needle was inserted into the subarachnoid space of the spinal cord between the L4 and L5 spinous processes. A quick tail flick indicated successful intrathecal injection. The injection itself did not alter the animal’s response time compared with measurements taken before the injection.

### Cell culture and focal repetitive magnetic stimulation treatment

*In vitro* tests were conducted using BV-2 cells (a microglial cell line, EK-Bioscience, Shanghai, China, Cat# CC-Y2022, RRID: CVCL_5I31). The cells were cultured in a humidified atmosphere of 5% CO_2_ at 37°C in Dulbecco’s modified Eagle’s medium (KenGEN BioTECH, Nanjing, China) supplemented with 10% (v/v) fetal bovine serum (Biological Industries, Jerusalem, Israel), 80 U/mL penicillin, and 0.08 mg/mL streptomycin. For further experiments, 1 × 10^5^ cells were seeded in a 6-well plate overnight.

BV-2 cells were pretreated with lipopolysaccharide (LPS; 1 µg/mL) for 12 hours before a single administration of focal rMS (10 Hz, 0.035 T, 1200 pulses). BV-2 cells were pretreated with STO-609 (1 μg/mL; MedChemExpress, Cat# HY-19805), a calmodulin-dependent protein kinase kinase beta (CaMKKβ) inhibitor, for 30 minutes.

### Transfection

For *in vivo* experiments, SOCS3 siRNA (Santa Cruz Biotechnology, Cat# SC-270156) was administered using *in vivo*-jetPEI® (Polyplus-transfection, Strasbourg, France, Cat# 101000040). SOCS3 siRNA (10 μmol) was dissolved in 1 mL of the *in vivo*-jetPEI:10% glucose mixture and intrathecally injected at 500 pmol/10 μL per rat. SOCS3 siRNA (Santa Cruz Biotechnology, Cat# SC-41001) was transfected into cells cultured in six-well plates with an antibiotic-free medium the day before transfection. Then, 3.3 nmol siRNA was dissolved in 330 μL RNase-free water. Transfection was conducted when cells reached 50%–70% confluence using Lipofectamine 2000 (Invitrogen, Carlsbad, CA, USA) and serum-free medium according to the manufacturer’s instructions. After 4 hours, the transfection medium was replaced with a culture medium containing 10% fetal bovine serum and then incubated at 37°C in 5% CO_2_.

### Western blotting

Samples (cells or spinal cord tissue segments at L4–L5; all rats were anesthetized with an intraperitoneal injection of 1% [w/v] pentobarbital sodium [50 mg/kg; Sigma-Aldrich, St. Louis, MO, USA]) and sacrificed through neck-breaking) were collected and washed with ice-cold phosphate-buffered saline and lysed in radioimmunoprecipitation assay lysis buffer. The lysates were separated by sodium dodecyl sulfate–polyacrylamide gel electrophoresis and electrophoretically transferred onto polyvinylidene fluoride membranes (Millipore, Bedford, MA, USA). The membranes were blocked with 10% low-fat dry powdered milk or 5% bovine serum albumin (Beyotime Biotechnology, Shanghai, China) and 5% low-fat dry powdered milk in Tris-buffered saline with Tween 20 for 2 hours at room temperature and then incubated with primary antibodies at 4°C overnight. Finally, the membranes were incubated with horseradish peroxidase-conjugated secondary antibodies (1:5000; Santa Cruz Biotechnology, Cat# sc-2357, RRID: AB_628497) for 2 hours at room temperature. The primary antibodies used included β-actin (rabbit, 1:5000; Abclonal, Wuhan, China, Cat# AC026, RRID: AB_2768234), SOCS3 (rabbit, 1:1000; Abclonal, Cat# A0694, RRID: AB_2757345), SOCS1 (rabbit, 1:1000; Abclonal, Cat# A7754, RRID: AB_2772339), CaMKKβ (rabbit; Cell Signaling Technology, Danvers, MA, USA, Cat# 16810, RRID: AB_2798771), phospho-CaMKKβ(Ser511) (rabbit, 1:500; Cell Signaling Technology, Cat# 12818, RRID: AB_2798034), phosphorylated AMPK (Thr172) (p-AMPK; rabbit, 1:1000; Cell Signaling Technology, Cat# 2535S, RRID: AB_331250), AMPK (rabbit, 1:2000; Abcam, Cambridge, UK, Cat# ab32047, RRID: AB_722764), glial fibrillary acidic protein (GFAP; rabbit, 1:1000; Cell Signaling Technology, Cat# 12389S, RRID:AB_2631098), ionized calcium binding adaptor molecule-1 (Iba-1; goat, 1:1000; Abcam, Cat# ab48004, RRID: AB_870576), matrix metalloproteinase 9 (MMP-9; rabbit,1:1000; Proteintech, Rosemont, IL, USA, Cat# 10375-2-AP, RRID: AB_2919732), phospho-signal transducer and activator of transcription 3 (rabbit, 1:1000; Abclonal, Cat# AP0070, RRID: AB_2771569), signal transducer and activator of transcription 3 (STAT3; rabbit, 1:1000; Abclonal, Cat# A1192, RRID: AB_2861642), phospho-p38 mitogen-activated protein kinase (p-p38; rabbit, 1:1000; Cell Signaling Technology; Cat# 4511, RRID: AB_2139682), p38 mitogen-activated protein kinase (p38; rabbit, 1:1000; Cell Signaling Technology, Cat# 4511, RRID: AB_11178801), TNF-α (1:1000; Abclonal, Cat# A20851), IL-1β (1:1000; Abclonal, Cat# A16288), and IL-6 (rabbit, 1:1000; Abcam, Cat# ab6672, RRID: AB_2127460). The bands were subsequently developed using enhanced chemiluminescence reagents (PerkinElmer, Waltham, MA, USA). Data were analyzed with Molecular Imager and the associated software Quantity One-4.6.5 (Bio-Rad Laboratories, Hercules, CA, USA).

### Immunofluorescence staining

The spinal cords (L4–L6) were removed and postfixed with a 4% paraformaldehyde solution for 24 hours at room temperature and a 30% sucrose solution for 24 hours at 4°C. The samples were frozen and sectioned to a thickness of 5 μm using a cryostat (CM1900 UV; Leica Microsystems GmbH, Wetzlar, Germany) for subsequent immunofluorescence analysis. Sections from each group were incubated with primary antibodies against Iba-1 (goat, 1:250; Wako, Osaka, Japan, Cat# 011-27991, RRID: AB_2935833), GFAP (goat, 1:200; Abcam, Cat# ab302644, RRID: AB_3669042), NeuN (E4M5P) (1:400; Cell Signaling Technology, Cat# 94403), c-Fos (mouse, 1:300; Abcam, Cat# ab208942, RRID: AB_2747772), calcitonin gene-related peptide (CGRP; rabbit, 1:400; Cell Signaling Technology, Cat# 14959, RRID: AB_2798662), and SOCS3 (rabbit, 1:50; Abmart, Shanghai, China, Cat# TD6133, RRID: AB_3669043) overnight at 4°C. They were washed and incubated for 2 hours at room temperature with appropriate secondary antibodies. The following secondary antibodies from Jackson Immunoresearch Laboratories (West Grove, PA, USA) were used: Alexa Fluor 488 AffiniPure donkey anti-rabbit immunoglobulin G (IgG) (H+L), Cy3 AffiniPure donkey anti-mouse IgG (H+L), and Cy3 AffiniPure donkey anti-goat IgG (H+L) (all 1:300; Jackson Immunoresearch Laboratories, Cat# 711-545-152/715-165-150/705-165-003, RRID: AB_2313584/AB_2340813/AB_2340411). The samples were investigated with a confocal microscope (LSM710; Zeiss, Oberkochen, Germany). The average numbers of microglia (Iba-1) and astrocytes (GFAP), and the average fluorescence intensity of c-Fos, and CGRP, in dorsal horn lamina I–III of the right spinal cord were analyzed using ImageJ software (version 1.53, National Institutes of Health, Bethesda, MD, USA) (Schneider et al., 2012) and normalized to the intensity levels of the Control group. The percentage of SOCS3-positive microglia and astrocytes in dorsal horn lamina I–III of the right spinal cord was determined by ImageJ software. Data were recorded from three random areas of lamina I–III from each dorsal horn section.

### Nuclear factor-kappa B activation assay

BV-2 cells were plated in glass-bottom cell culture dishes and treated with LPS for 12 hours, with or without focal rTSMS. Subsequently, BV-2 cells were fixed with 4% paraformaldehyde and permeated with 0.3% Triton X-100. This process was followed by blocking with 10% donkey serum in phosphate-buffered saline for 2 hours. Coverslips with BV-2 cells were incubated at 4°C with the nuclear factor-kappa B (NF-κB) p65 antibody (rabbit; Abmart, Cat# T55034, RRID:AB_2937049), diluted in phosphate-buffered saline (1:50) overnight, and exposed to fluorescein isothiocyanate-conjugated anti-rabbit IgG (1:300, room temperature for 1 hour). They were then rinsed three times with phosphate-buffered saline and stained with 1 μg/mL 4′,6-diamidino-2-phenylindole (a fluorescence DNA dye to mark the nucleus; Beyotime Biotechnology) for 1 minute. Confocal microscopy analysis was conducted using the Zeiss LSM710 confocal system.

### Statistical analysis

All the statistical analyses were conducted using GraphPad Prism software (version 9.0 c; GraphPad Software, Inc., La Jolla, CA, USA). All data are presented as the mean ± standard deviation of independent experiments. Repeated-measures two‐way analysis of variance was used to analyze the effects of time and repeated treatments on focal rTSMS. The differences between time points within groups, and between each group within the same time point, were also analyzed with one-way analysis of variance. Differences among groups were analyzed by one-way analysis of variance followed by Tukey’s multiple comparisons tests after normality and homoscedasticity tests. *P* < 0.05 was considered statistically significant.

## Results

### Single or repetitive administration of focal repetitive trans-spinal magnetic stimulation dramatically attenuates chronic constriction injury-induced mechanical pain sensitivity

**Figure 1:**
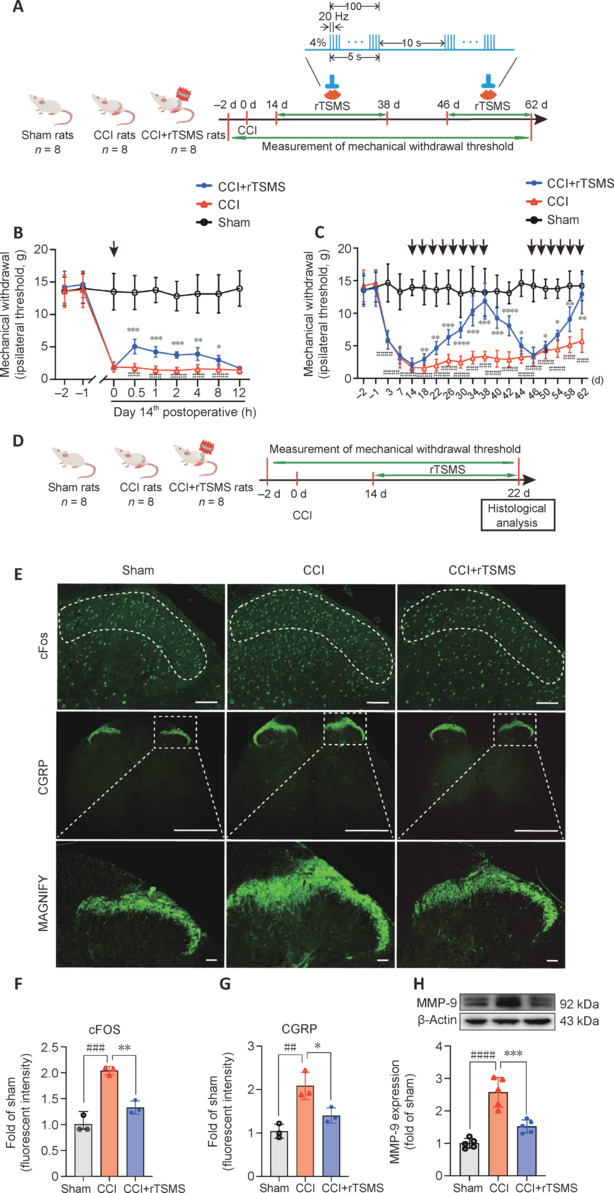
Focal rTSMS attenuates CCI-induced mechanical pain sensitivity. (A) A diagram showing the timeline of CCI modeling, rTSMS treatment, and mechanical withdrawal threshold assessment in rats. (B) A single administration of focal rTSMS significantly attenuated CCI-induced mechanical pain sensitivity (*n* = 8). Arrow indicates rTSMS intervention. (C) Re-administration of focal rTSMS significantly suppressed CCI-induced mechanical pain sensitivity (*n* = 8). Arrow indicates rTSMS intervention. (D) A diagram showing the timeline of CCI modeling, rTSMS treatment, and experimental analysis in rats. (E–G) Representative immunofluorescence images and quantitative analysis showing that re-administration of rTSMS significantly inhibited CCI-induced upregulation of c-Fos and CGRP in the dorsal horn of the spinal cord (*n* = 3). Dashed box indicates the region of interest in the dorsal horn of the spinal cord. Scale bars: 100 μm. (H) Western blotting data indicated that re-administration of rTSMS significantly inhibited CCI-induced upregulation of MMP-9 in the spinal cord (*n* = 5). Data are expressed as mean ± SD. ##*P* < 0.01, ###*P* < 0.001, ####*P* < 0.0001, *vs*. Sham group; *P < 0.05, **P < 0.01, ****P < 0.0001, *vs.* CCI group (B, C: two‐way analysis of variance; E–H: one‐way analysis of variance followed by Tukey’s multiple comparisons tests). CCI: Chronic constrictive injury; CGRP: calcitonin gene-related peptide; MMP-9: matrix metalloproteinase 9; rTSMS: repetitive trans-spinal magnetic stimulation.

A CCI model was used to investigate the analgesic effect of focal rTSMS on NP. From day 14 of CCI modeling, rats were treated with focal rTSMS (20 Hz, 0.14 T, 4000 pulses) over the lumbosacral region (twice a day for 5 consecutive days followed a 2-day interval, and then twice a day for an additional 5 consecutive days). After the 24-day intervention, rats received no treatment for the next 8 days. Subsequently, rats received focal rTSMS 5 days per week for 16 consecutive days (**Figure 1A**). The mechanical withdrawal thresholds were reduced in the CCI group compared with the Sham group and remained at a lower level for a long time starting from day 14 of modeling. It was found that a single administration of focal rTSMS significantly attenuated CCI-induced mechanical allodynia, and the analgesic effect lasted for 8 hours (*P* < 0.001; **Figure 1B**). Furthermore, the analgesic effect continued after focal rTSMS re-administration. After 24 days of multiple administrations of focal rTSMS, rats in the CCI + rTSMS group exhibited a significant increase in the mechanical withdrawal threshold, close to that of the Sham group rats. However, the mechanical withdrawal thresholds of rats in the CCI + rTSMS group decreased gradually after the intervention and were close to those of the CCI rats without focal rTSMS treatment by day 8. Finally, the rats in the CCI + rTSMS group received another 16 days of focal rTSMS treatment, and the mechanical pain thresholds of these rats rebounded from 3.583 to 12.960 g on day 16 of the second intervention, close to those of the Sham group (**Figure 1C**). These behavioral data suggest that focal rTSMS can effectively alleviate mechanical pain sensitivity in rats with CCI-induced NP.

Previous studies demonstrated that c-Fos (a specific marker of neuronal excitability; Pan et al., 2018), CGRP, and MMP-9 in the spinal cord have a well-established role in central sensitization in NP (Pan et al., 2018; He et al., 2019). Subsequently, the expression levels of these biomarkers were evaluated in the spinal cords of rats in the three groups. As shown in **Figure 1E–H**, CCI significantly elevated the levels of c-Fos, CGRP, and MMP-9 in the spinal cords (*P* < 0.001), consistent with the pattern of occurrence of NP-related behaviors in rats. However, 8 consecutive days of focal rTSMS treatment significantly inhibited these biomarkers in the spinal cord (*P* < 0.05). Taken together, these behavioral and molecular biological data suggest that focal rTSMS can effectively relieve CCI-induced mechanical pain sensitivity (**Figure 1D**).

### Focal repetitive trans-spinal magnetic stimulation inhibits chronic constriction injury-induced neuroinflammation and upregulates SOCS3 in the spinal cord

**Figure 2:**
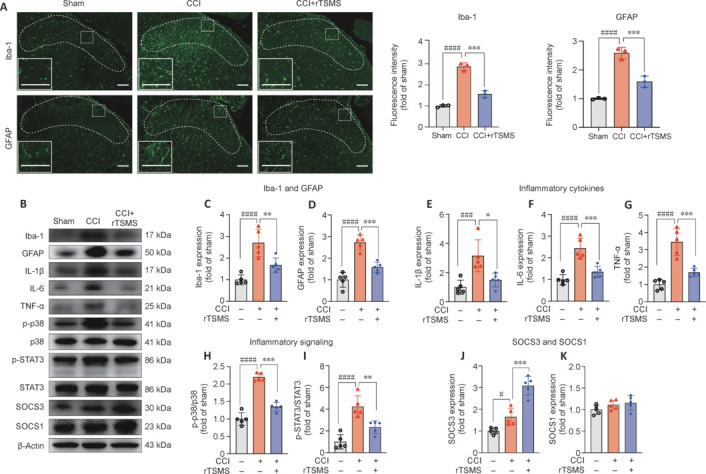
Focal rTSMS significantly inhibits CCI-induced neuroinflammation and upregulates SOCS3 in the spinal cord *in vivo*. (A) Representative immunofluorescence images showing the inhibitory effects of focal rTSMS on CCI-induced activation of microglia and astrocytes in the dorsal horn (*n* = 3). Dashed box indicates the region of interest in the dorsal horn of the spinal cord. Scale bars: 100 μm. (B) Representative image of western blotting. (C, D) Re-administration of rTSMS significantly inhibited the CCI-induced upregulation of Iba-1 and GFAP (*n* = 5). (E–G) Focal rTSMS significantly inhibited CCI-induced upregulation of IL-1β, IL-6, and TNF-α in the spinal cord (*n* = 5). (H, I) Focal rTSMS significantly inhibited the CCI-induced increase in the protein levels of p-p38 and p-STAT3 in the spinal cord (*n* = 5). (J) Focal rTSMS significantly upregulated SOCS3 in the spinal cord (*n* = 5). (K) Focal rTSMS could not upregulate SOCS1 in the spinal cord (*n* = 5). Data are expressed as mean ± SD. #*P* < 0.05, ###*P* < 0.001, ####*P* < 0.0001, *vs.* Sham group; *P < 0.05, **P < 0.01, ***P < 0.001, *vs*. CCI group (one‐way analysis of variance followed by Tukey’s multiple comparisons tests). CCI: Chronic constrictive injury; GFAP: glial fibrillary acidic protein; Iba-1: ionized calcium binding adaptor molecule-1; IL: interleukin; rTSMS: repetitive trans-spinal magnetic stimulation; SOCS: suppressor of cytokine signaling; STAT3: transducer and activator of transcription 3; TNF-α: tumor necrosis factor-alpha.

Accumulating evidence indicates that neuroinflammation, mainly caused by glial activation, especially microglial activation, plays a key role in maintaining central sensitization in NP (Yi et al., 2021). Thus, immunofluorescence and western blotting assays were performed to investigate the effects of focal rTSMS on CCI-induced neuroinflammation *in vivo*. The results revealed a significant increase in the levels of Iba-1 (a microglial marker; Qian et al., 2023) and GFAP (an astrocyte marker; Qian et al., 2023) in the spinal cord of CCI rats, indicating the activation of microglia and astrocytes in the spinal cord, with the increase being reversed by the administration of focal rTSMS (**Figure 2A–D**). Furthermore, the protein levels of the proinflammatory cytokines IL-1β, IL-6, and TNF-α were analyzed to determine the anti-inflammatory effect of focal rTSMS. Western blotting analysis revealed that focal rTSMS remarkably inhibited CCI-induced upregulation of IL-1β, IL-6, and TNF-α (**Figure 2B** and **E–G**). Accumulating evidence indicates that the p38 (MAPK) and STAT3 pathways contribute to pain sensitization after nerve injury by inducing neuroinflammation (Liu et al., 2020; Qian et al., 2023). Our data showed that CCI activated p38 and STAT3, which were inhibited by rTSMS (**Figure 2B**, **H** and **I**). These data indicate that focal rTSMS can inhibit neuroinflammation in the spinal cord of CCI rats. To investigate the mechanism underlying the anti-neuroinflammatory effects of focal rTSMS in the spinal cord, we investigated suppressor of cytokine signaling (SOCS), an endogenous negative regulator of cytokine signaling in different diseases (Wan et al., 2022; Qian et al., 2023). As shown in **Figure 2B**, **J** and **K**, focal rTSMS significantly increased the protein level of SOCS3 in the spinal cord (*P* < 0.001) but it could not increase the protein level of SOCS1. Therefore, we speculate that the anti-neuroinflammatory effects of focal rTSMS in the spinal cord might depend on SOCS3.

### Focal repetitive trans-spinal magnetic stimulation requires SOCS3 to attenuate chronic constriction injury-induced mechanical pain sensitivity in rats *in vivo*

**Figure 3:**
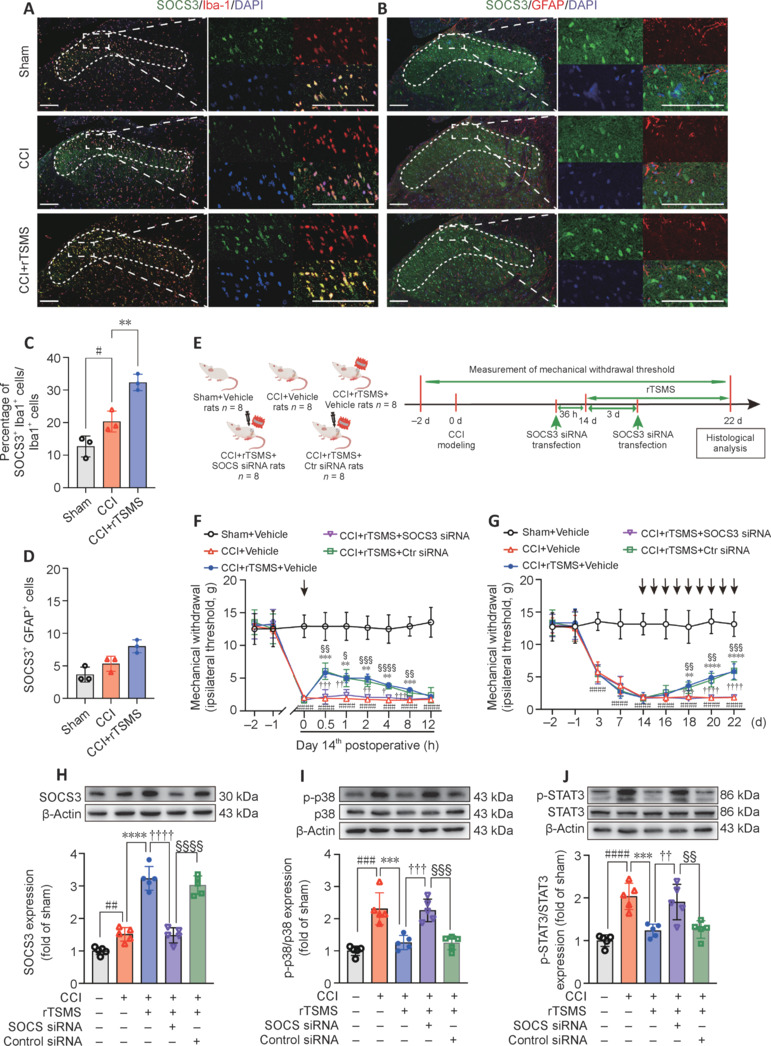
SOCS3 mediates the effects of focal rTSMS in attenuating CCI-induced mechanical pain sensitivity in rats *in vivo*. (A–D) Representative immunofluorescence images showing that focal rTSMS increased SOCS3 in the microglia, but not in astrocytes (*n* = 3). Representative confocal microscopic images indicating the expression of SOCS3 (green, Alexa Fluor 488) in microglia (Iba‐1, red, Cy3) and astrocytes (GFAP, red, Cy3) in the dorsal horn in the spinal cord. Scale bars: 100 μm. (E) A timeline diagram of CCI modeling, rTSMS treatment, mechanical withdrawal threshold assessment, and experimental analysis in rats. (F, G) Intrathecal injection of SOCS3 siRNA significantly abrogated the alleviation of mechanical allodynia by single or repeated administration of focal rTSMS after CCI (*n* = 8). (H) Western blotting data showing that intrathecal injection of SOCS3 siRNA significantly reversed focal rTSMS-induced upregulation of SOCS3 in the spinal cord (*n* = 5). (I, J) Western blotting results showing that intrathecal injection of SOCS3 siRNA significantly reversed the focal rTSMS-induced decrease in protein levels of phosphorylated p38 and STAT3 in the spinal cord (*n* = 5). Data are expressed as mean ± SD. #*P* < 0.05, ##*P* < 0.01, ###*P* < 0.001, ####*P* < 0.0001, *vs.* Sham + Vehicle group; **P < 0.01, ****P < 0.0001, *vs*. CCI + Vehicle group; †*P* < 0.05, ††*P* < 0.01, †††*P* < 0.001, ††††*P* < 0.0001, *vs.* CCI + rTSMS + Vehicle group; §*P* < 0.05, §§*P* < 0.01, §§§*P* < 0.001, §§§§*P* < 0.0001, *vs*. CCI + rTSMS + SOCS3 siRNA group (F, G: two‐way analysis of variance; C, D, H–J: one‐way analysis of variance followed by Tukey’s multiple comparisons tests). CCI: Chronic constrictive injury; GFAP: glial fibrillary acidic protein; Iba-1: ionized calcium binding adaptor molecule-1; rTSMS: repetitive trans-spinal magnetic stimulation; SOCS3: suppressor of cytokine signaling 3; STAT3: transducer and activator of transcription 3.

Confocal microscopic scanning was performed in the spinal cord to analyze SOCS3 expression after focal rTSMS treatment. The colocalization of SOCS3 with Iba-1 increased dramatically in spinal cord lamina I–III after rTSMS treatment; however, the colocalization of SOCS3 with GFAP did not significantly change (*P* < 0.01; **Figure 3A–D**). In addition, SOCS3 was also co-labeled with neurons in the spinal dorsal horn; however, the colocalization of SOCS3 with neurons did not change significantly (*P* < 0.01; **Additional Figure 1A** and **B**). We then explored whether SOCS3 is required for focal rTSMS-mediated attenuation of CCI-induced mechanical pain sensitivity, and whether SOCS3 knockdown in the spinal cord could reverse the analgesic effect of focal rTSMS. SOCS3 siRNA was intrathecally injected to downregulate SOCS3 (**Figure 3E**). As anticipated, intrathecal injection of SOCS3 siRNA significantly reversed focal rTSMS-induced upregulation of SOCS3 in the spinal cord and abrogated focal rTSMS-induced alleviation of mechanical allodynia after CCI modeling (*P* < 0.001; **Figure 3F–H**). Moreover, focal rTSMS did not inhibit the CCI-induced phosphorylation of p38 and STAT3 after SOCS3 knockdown, further indicating that the anti-neuroinflammatory effects of focal rTSMS in the spinal cord might depend on SOCS3 (**Figure 3I** and **J**). Thus, rTSMS-induced attenuation of mechanical allodynia in CCI rats depends on SOCS3 upregulation in the microglia.

### Repetitive magnetic stimulation significantly suppresses the SOCS3-dependent inflammatory response in microglia *in vitro*

**Figure 4:**
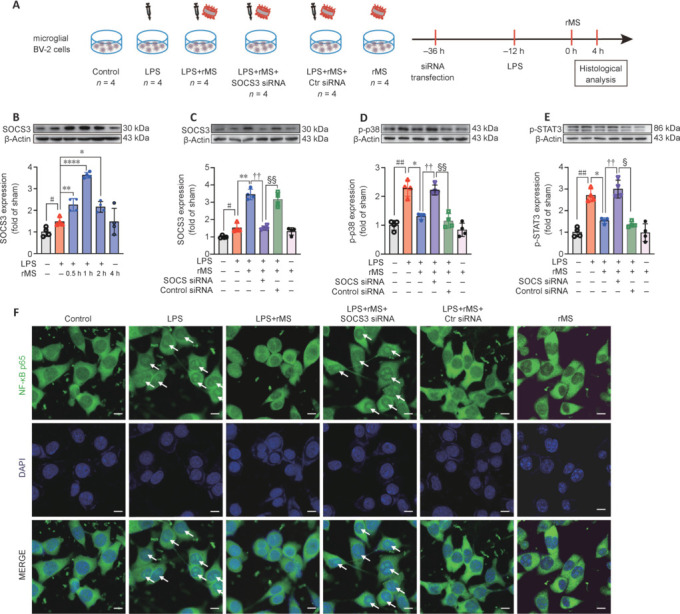
rMS suppresses the inflammatory response in a SOCS3-dependent manner in microglia *in vitro*. (A) A schematic diagram of the *in vitro* study. (B) Western blotting data showing that the SOCS3 protein level in BV-2 cells *in vitro* increased at 0.5 hours after a single administration of rMS and was highest at 1-hour post-rMS (*n* = 4). (C) Western blotting analysis indicating that SOCS3 siRNA significantly reversed focal rMS-induced upregulation of SOCS3 *in vitro* (*n* = 4). (D, E) The expression of phosphorylated p38 and STAT3 in the spinal cord following SOCS3 siRNA transfection and rMS treatment, as determined by Western blotting (*n* = 4). (F) Representative immunofluorescence images showing that rMS inhibited the translocation of NF-κB p65 (green, Alexa Fluor 488) from the cytosol to the nucleus after LPS treatment *in vitro*, and this effect was reversed by administration of SOCS3 siRNA (100 pmol/500 μL) 36 hours before LPS pretreatment (*n* = 3). Arrows indicate BV-2 cells. Scale bars: 10 μm. Data are expressed as mean ± SD. #*P* < 0.05, ##*P* < 0.01, *vs.* Control group; *P < 0.05, **P < 0.001, ****P < 0.001, *vs*. LPS group; ††*P* < 0.01, *vs.* LPS + rMS group; §*P* < 0.05, §§*P* < 0.01, *vs*. LPS + rTSMS + SOCS3 siRNA group (one‐way analysis of variance followed by Tukey’s multiple comparisons tests). DAPI: 4′,6-Diamidino-2-phenylindole; LPS: lipopolysaccharide; NF-κB: nuclear factor kappa-B; rMS: repetitive magnetic stimulation; SOCS3: suppressor of cytokine signaling 3; STAT3: transducer and activator of transcription 3.

An immortalized murine microglial BV-2 cell line was used to uncover the potential relationship between focal rTSMS and neuroinflammation in the spinal cord. B-V2 cells were pretreated with LPS for 12 hours before a single administration of focal rMS (**Figure 4A**). As shown in **Figure 4B**, the SOCS3 protein level increased at 0.5 hours in B-V2 cells *in vitro* compared with the LPS group after a single administration of focal rMS, was highest at 1 hour post-rMS, but decreased at 4 hours post-rMS, with no significant increase in SOCS3 protein expression. SOCS3 siRNA (100 pmol/500 μL) was used to downregulate SOCS3 in the microglial cell line *in vitro* to investigate whether the anti-inflammatory effects of rMS were microglial SOCS3-dependent. Western blotting analysis revealed that SOCS3 siRNA abrogated the effect of rMS on SOCS3 upregulation (**Figure 4C**). In addition, SOCS3 knockdown sufficiently abrogated rMS-induced inhibition of p38 and STAT3 phosphorylation (**Figure 4D** and **E**). Previous studies demonstrated that the activation of p38/NF-κB signaling in microglia leads to a burst in neuroinflammation (Wang et al., 2020). We found that LPS increased the phosphorylation of p38 (**Figure 4D**) and caused the translocation of NF-κB p65 from the cytoplasm to the nucleus (**Figure 4F**). rMS markedly suppressed p38 phosphorylation and inhibited NF-κB p65 nuclear translocation in LPS-pretreated BV-2 cells, whereas these effects were reversed by SOCS3 siRNA treatment (**Figure 4D** and **F**). Collectively, these data demonstrated that rMS relieves neuroinflammation by inducing the upregulation of microglial SOCS3 to inhibit the phosphorylation of p38 and STAT3, and NF-κB p65 nuclear translocation.

### Focal repetitive trans-spinal magnetic stimulation-induced SOCS3 upregulation is CaMKKβ/AMPK-dependent

**Figure 5:**
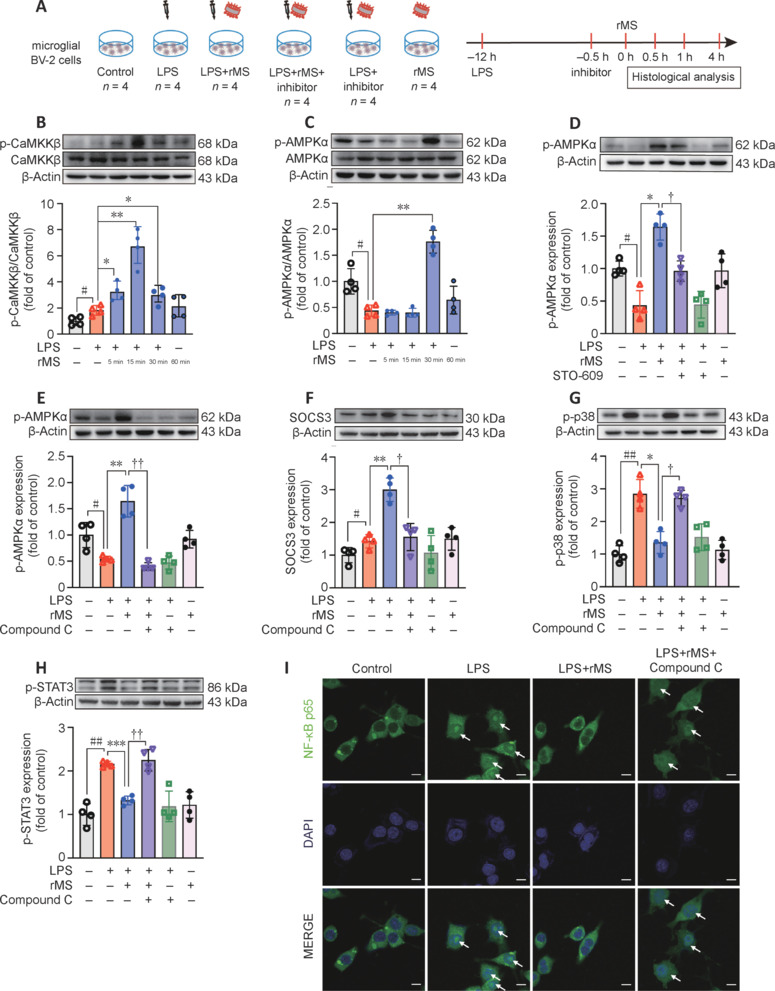
rMS-induced upregulation of SOCS3 in microglia is CaMKKβ/AMPK-dependent *in vitro*. (A) A schematic diagram of the *in vitro* study. (B) Western blotting data indicating that the p-CaMKKβ protein level in BV-2 cells increased at 5 minutes after a single administration of rMS and was highest at 15 minutes post-rMS (*n* = 4). (C) Western blotting data showing that the p-AMPK protein level in BV-2 cells increased significantly at 30 minutes after rMS (*n* = 4). (D, E) Representative western blotting results show that STO-609 and compound C alleviated rMS-induced activation of AMPK *in vitro* (*n* = 4). (F–H) Representative western blotting data showing that compound C abrogated rMS-induced upregulation of SOCS3 and inhibition of p38 and STAT3 *in vitro* (*n* = 4). (I) Representative immunofluorescence images showing that rMS inhibited the translocation of NF-κB p65 (green, Alexa Fluor 488) from the cytosol to the nucleus after LPS treatment *in vitro*, where this effect was reversed by administration of compound C (20 μmol/mL) 0.5 hours before LPS pretreatment (*n* = 3). Arrows indicate BV-2 cells. Scale bars: 10 μm. Data are expressed as mean ± SD. #*P* < 0.05, ##*P* < 0.01, *vs*. Control group; *P < 0.05, **P < 0.01, ****P < 0.001, *vs*. LPS group; †*P* < 0.05, ††*P* < 0.01, *vs.* LPS + rMS group (one‐way analysis of variance followed by Tukey’s multiple comparisons tests). AMPK: Adenosine 5′-monophosphate-activated protein kinase; CaMKKβ: calmodulin-dependent protein kinase kinase beta; Compound C: an AMPK inhibitor; DAPI: 4′,6-diamidino-2-phenylindole; LPS: lipopolysaccharide; NF-κB: nuclear factor kappa-B; p-AMPK: phosphorylated AMPK(Thr172); p-CaMKKβ: phospho-CaMKKβ(Ser511); rMS: repetitive magnetic stimulation; SOCS3: suppressor of cytokine signaling 3; STAT3: transducer and activator of transcription 3; STO-609: a CaMKKβ inhibitor.

AMPK is a key cellular energy sensor and master regulator of energy homeostasis, and its activation relieves different types of pain (Asiedu et al., 2016; Xiang et al., 2019; Zheng et al., 2021). We speculated that the bioeffects of magnetic stimulation in CCI models might be induced by AMPK activation. Hence, the protein expression of AMPK and its upstream CaMKKβ were detected in BV-2 cells *in vitro* to uncover the correlation between focal rTSMS and AMPK. As shown in **Figure 5B**, the protein level of phospho-CaMKKβ(Ser511) increased slightly 5 minutes after rMS treatment, peaked at 15 minutes, declined at 30 minutes, and was close to the Control group level at 60 minutes, suggesting that rMS can activate CaMKKβ in microglia. In addition, phosphate-AMPK-alpha strikingly increased 30 minutes after rMS treatment, whereas pretreatment with STO-609 abolished AMPK activation by rMS (**Figure 5C** and **D**). Therefore, rMS can activate AMPK in microglia. Our previous study demonstrated that AMPK activation induces microRNA-30a-5p inhibition to directly target SOCS3 (Wan et al., 2022). The present study found that administration of compound C (20 μmol/mL) 0.5 hours before rMS inhibited rMS-induced AMPK activation, SOCS3 upregulation, and p38 and STAT3 phosphorylation (**Figure 5E–H**). The immunofluorescence results showed that rMS could not inhibit NF-κB p65 nuclear translocation in LPS-pretreated BV-2 cells also pretreated with compound C (**Figure 5I**). *In vitro* studies showed that rMS-induced SOCS3-dependent neuroinflammatory suppression is related to the activation of CaMKKβ/AMPK signaling.

**Figure 6:**
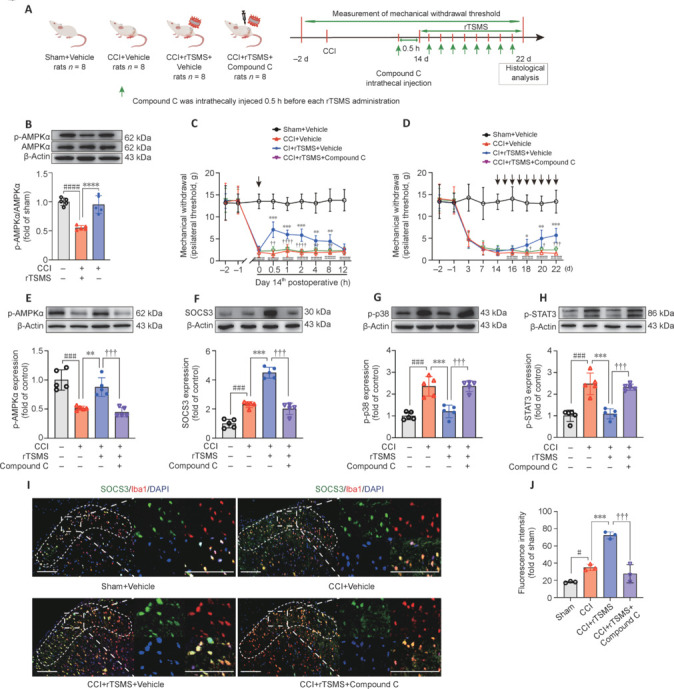
The pain-relieving effect and upregulation of SOCS3 induced by focal rTSMS depended on the activation of AMPK. (A) Timeline diagram of CCI modeling, rTSMS treatment, mechanical withdrawal threshold assessment, and experimental analysis in rats. (B) Western blotting data illustrates that the p-AMPK protein level decreased in CCI model rats and increased in CCI + rTSMS + Vehicle rats (*n* = 5). (C, D) Intrathecal injection of compound C largely prevented the pain-relieving effects of focal rTMS, when applied once or repeatedly, in rats with CCI neuropathic pain (*n* = 8). (E–H) Representative western blotting results showing that compound C alleviated the rTSMS-induced activation of AMPK, increase in SOCS3, and downregulation of p38 and STAT3 (*n* = 5). (I, J) Representative immunofluorescence images showing that compound C abrogated the rTSMS-induced upregulation of SOCS3 (green, Alexa Fluor 488) in the dorsal horn of the spinal cord (*n* = 3). Dashed box indicates the region of interest in the dorsal horn of the spinal cord. Scale bars: 100 μm. Data are expressed as mean ± SD. #*P* < 0.05, ##*P* < 0.01, ###*P* < 0.001, *vs.* Sham + Vehicle group; *P < 0.05, **P < 0.01, ****P < 0.0001, *vs*. CCI + Vehicle group; †*P* < 0.05, ††*P* < 0.01, †††*P* < 0.001, ††††*P* < 0.0001, *vs*. CCI + rTSMS + Compound C group (C, D: two‐way analysis of variance; B, E–H, J: one‐way analysis of variance followed by Tukey’s multiple comparisons tests). AMPK: Adenosine 5′-monophosphate-activated protein kinase; CCI: chronic constrictive injury; DAPI: 4′,6-diamidino-2-phenylindole; Iba-1: ionized calcium binding adaptor molecule-1; p-AMPK: phosphorylated AMPK(Thr172); p-p38: phospho-p38 mitogen-activated protein kinase; p-STAT3: phospho-STAT3; rTSMS: repetitive trans-spinal magnetic stimulation; SOCS3: suppressor of cytokine signaling 3; STAT3: transducer and activator of transcription 3.

To further validate the *in vitro* results, we performed an *in vivo* experiment to assess whether the SOCS3-dependent analgesic effect of focal rTSMS in the CCI model was dependent on AMPK activation (**Figure 6A–J**). The phosphate-AMPK-alpha/AMPKα ratio in the spinal cord decreased after CCI modeling, whereas focal rTSMS exhibited stronger AMPK-activation in the spinal cords of CCI-treated rats (**Figure 6B**). Meanwhile, intrathecal injection of compound C (10 µg/10 µL) 0.5 hours before focal rTSMS abrogated the rTSMS-induced mechanical allodynia alleviation in the CCI model (**Figure 6C** and **D**). These results demonstrated that the alleviation of mechanical allodynia by focal rTSMS depended on AMPK activation. Furthermore, consistent with the *in vitro* results, intrathecal injection of compound C before focal rTSMS abolished focal rTSMS-induced AMPK activation, microglial SOCS3 upregulation, and phosphorylation of p38 and STAT3 (**Figure 6E–J**). Together, these *in vitro* and *in vivo* findings support our hypothesis that focal rTSMS-induced SOCS3-dependent neuroinflammatory suppression and mechanical allodynia alleviation in CCI rats occurred through activation of the CaMKKβ/AMPK pathway.

## Discussion

### Focal repetitive trans-spinal magnetic stimulation effectively ameliorates chronic constriction injury-induced mechanical pain sensitivity and inhibits neuroinflammation in the spinal cord of chronic constriction injury rats

It is well-accepted that neuroinflammation in the spinal cord, characterized by the glial release of proinflammatory cytokines, plays a crucial role in the induction and maintenance of NP (Bouhassira, 2019; Di et al., 2025). Accumulating preclinical studies have indicated that glial cell activation and glia–neuron interactions are involved in the pathogenesis of chronic pain because of their contributions to neuroinflammation and the resulting alternations in neuronal functions (Malcangio, 2019; Dou et al., 2021; Luo et al., 2021). Particularly, p38 MAPK has emerged as an important glial intracellular mediator (Ma and Quirion, 2005; Ji and Suter, 2007). In NP models, damage-associated molecular pattern molecules released by damaged peripheral nerves bind to microglial TLR4 to target the TLR4/p38MAPK/NF-κB pathway in the spinal cord. Then, microglia, the principal immune cells, transform from a resting to an activated state and release a repertoire of proinflammatory factors, including IL-1β, IL-6, and TNF-α, which cause extracellular-regulated kinase-dependent potentiation of N-methyl-D-aspartate currents in dorsal horn neurons, leading to synaptic plasticity and central sensitization pain (Sommer et al., 2018; Hu et al., 2020; Qiu et al., 2021). In addition, these proinflammatory factors can bind to the IL-1 receptor, IL-6 receptor, or TNF receptor of microglia, leading to intracellular cascades and aggravation of neuroinflammation (Sommer et al., 2018). For instance, IL-6 is involved in the activation of Janus kinase 2/STAT3 signaling, inducing proinflammatory responses (Li et al., 2022). Moreover, these proinflammatory factors can activate astrocytes to reinforce pain signaling (Linnerbauer et al., 2020). It is reported that microglia are indispensable for chronic pain-related synaptic plasticity in the spinal dorsal horn (Zhou et al., 2019). Although direct targeting of microglial activation and neuroinflammation via microglial inhibitors, TLRs, proinflammatory factors, and MAPKs may be effective, they are characterized by side effects such as infection and impairment of the resolution of inflammation (Chen et al., 2017). However, there is currently no noninvasive anti-inflammatory analgesic strategy targeting the spinal cord for the clinical treatment of NP. Therefore, there is an urgent need to identify emerging noninvasive and nondrug medical therapies with promise for the modulation of homeostasis of the central nervous system and attenuation of NP.

Bioelectromagnetic field therapy has been used in the treatment of different types of pain. The control of pain exerted by glial cells in the spinal cord has emerged as a promising target against NP in recent years. Our previous study showed that focal rTSMS decreased inflammation in SCI animal models (Zhai et al., 2024). It seems that rTSMS is an effective anti-neuroinflammatory option. Thus, we speculated that focal rTSMS might be an effective intervention for NP relief. Our study revealed a reduction in mechanical pain intensity and c-Fos, CGRP, and MMP-9 levels after focal rTSMS, verifying our conjecture. c-Fos and CGRP are markers of neural excitability. Our results demonstrated that, following CCI, c-Fos and CGRP significantly increased. Conversely, following rTSMS treatment, c-Fos and CGRP activation was significantly inhibited in CCI rats, thereby verifying the hypothesis that rTSMS treatment inhibits neural excitability. Additionally, the inhibitory effect of rTSMS on neural excitability can be corroborated through the use of neurophysiological techniques.

rTMS has been increasingly studied in different neurological diseases in the past decade. Although most studies focus on the effects of rTMS on neuronal cells, a contribution of non-neuronal cells to the rTMS-triggered improvement in these diseases is increasingly being suggested. Mounting studies have reported that the effects of rTMS are involved in anti-inflammation in different animal disease models (Stampanoni Bassi et al., 2020; Clarke et al., 2021; Toledo et al., 2021a). One study reported that the anti-inflammatory actions exerted by rTMS were associated with the TLR4/NF-κB/NLRP3 signaling pathway in mice with chronic unpredictable mild stress-induced depression (Zuo et al., 2022). Furthermore, our previous study showed that high-frequency rTMS protected against cerebral ischemia/reperfusion injury in rats by mitigating ferroptosis and inflammation (Zhou et al., 2023). In addition, rTMS improved neurological function and inhibited neuroinflammation in ischemic rats (Luo et al., 2022a). Consistently, the present study found that focal rTSMS inhibited the activation of microglia and astrocytes, the release of proinflammatory cytokines, and the phosphorylation of p38 and STAT3 in the spinal cord of CCI-induced NP rats. *In vitro* experiments showed that rMS inhibited NF-κB p65 nuclear translocation. All the results indicated that rTSMS is an effective anti-neuroinflammatory strategy, and focal rTSMS-induced mechanical pain relief might be related to the inhibition of glial activation and neuroinflammation suppression.

### Focal repetitive trans-spinal magnetic stimulation-induced analgesic effect and neuroinflammation inhibition depend on SOCS3 upregulation in microglia in the spinal cord

Next, we investigated how focal rTSMS modulates neuroinflammation in the spinal cord. SOCS family proteins play a key role in the negative regulation of cytokine signaling in immunity (Baker et al., 2009). Among the SOCS family proteins, SOCS1 and SOCS3 participate in the control of central nervous system immunity and modulation of neuroinflammation-mediated diseases (Cao et al., 2018; Yu et al., 2020b; Zhou et al., 2020). It was found that rTSMS upregulated SOCS3 but not SOCS1 in the spinal cord of CCI-induced NP rats. Thus, SOCS3 might be the key bio-mediator for rTSMS to effectively suppress neuroinflammation.

To validate this hypothesis, we performed *in vivo* and *in vitro* experiments, and the results showed that focal rTSMS increased SOCS3 in the microglia in the spinal cord but not in astrocytes. Additionally, SOCS3 knockdown in the spinal cord abrogated the analgesic effects of focal rTSMS and the inhibition of phosphorylation of p38 or STAT3 *in vivo*. These data suggested that focal rTSMS-induced analgesic effects and neuroinflammation suppression might depend on selective upregulation of SOCS3 in microglia.

Moreover, we performed *in vitro* experiments to gain more insight into the therapeutic effects of rTSMS at the cellular level. Consistent with the results of *in vivo* studies, focal rTSMS increased SOCS3 and inhibited p38 or STAT3 phosphorylation in the microglia. Furthermore, focal rTSMS inhibited the NF-κB translocation from the cytosol to the nucleus in microglia. Although reports showing that the focal rTSMS-induced anti-inflammatory effect depends on SOCS3 upregulation in microglia are scarce, a recent study found that rTMS induced a switch in microglia polarization from the proinflammatory phenotype to the anti-inflammatory phenotype (Zuo et al., 2022). SOCS3 has been considered an anti-inflammatory type macrophage/microglia marker (Huang et al., 2022). Therefore, it is reasonable to believe that focal rTSMS-induced anti-inflammation depends on the upregulation of SOCS3 in microglia. The current study reported for the first time that focal rTSMS relieves mechanical allodynia and neuroinflammation by inducing upregulation of microglial SOCS3, inhibiting the phosphorylation of p38 and STAT3 and NF-κB p65 nuclear translocation.

We also found that SOCS3 expression increased, whereas the number of Iba-1^+^ microglia decreased, after rTSMS treatment. We hypothesized that increased SOCS3 disrupts the inflammatory cascade, which is an important cause of microglial proliferation. Because the SOCS3 level is very low in the healthy state, we did not validate whether SOCS3 knockdown in healthy rats can induce mechanical allodynia.

### Focal repetitive trans-spinal magnetic stimulation-induced analgesic effect and SOCS3 upregulation in microglia in the spinal cord depend on adenosine 5′-monophosphate-activated protein kinase activation

Studies exploring the relationship between focal rTSMS and SOCS3 are limited. At present, the mechanism underlying the regulation of SOCS3 by rTSMS is unclear. AMPK, a pivotal cellular energy sensor and master regulator of energy homeostasis, is associated with pain (Lu et al., 2017). In vivo studies have reported that both electrical and magnetic therapy are associated with energy changes (Jauch-Chara et al., 2015; Chen et al., 2021). Moreover, our previous study showed that activation of AMPK could induce SOCS3 upregulation (Wan et al., 2022). Therefore, we hypothesized that SOCS3 upregulation by rTSMS to achieve analgesia might depend on the activation of AMPK. The present study found that CCI modeling inhibited AMPK activation in the spinal cord, focal rTSMS activated AMPK by phosphorylating on Thr172, and AMPK inhibition in the spinal cord ablated the analgesic effect of focal rTSMS. Further *in vivo* and *in vitro* studies revealed that AMPK inhibition also ablated SOCS3 upregulation and neuroinflammatory suppression in the spinal cord by rTSMS or rMS. Our previous studies also showed that activation of the AMPK–autophagy axis inhibited miRNA-30a-5p by degrading DICER and Argonaute 2 to upregulate SOCS3 (Wan et al., 2022). Collectively, these data suggest that the rTSMS-induced analgesic effect and SOCS3 upregulation in microglia in the spinal cord depend on the activation of AMPK. Focal rTSMS has bright prospects for the treatment of NP. The results also indicate that AMPK activation may be the future of bioelectromagnetic research.

### Focal repetitive trans-spinal magnetic stimulation-induced adenosine 5′-monophosphate-activated protein kinase activation depends on calmodulin-dependent protein kinase kinase beta activation

Our study found that rTSMS activated AMPK by phosphorylating on Thr172. AMPK can also be directly phosphorylated on Thr172 in response to calcium flux by the calcium-sensitive kinase CAMKKβ (Woods et al., 2005). Qiao et al. (2020) reported that curcumin prevents neuroinflammation via CaMKKβ-dependent activation of the AMPK signal pathway. Our *in vitro* results showed that focal rMS activated CaMKKβ in microglia. Furthermore, the CaMKKβ inhibitor STO-609 counteracted AMPK activation by focal rMS. These results indicated that rMS-induced AMPK activation depends on the activation of CAMKKβ. However, how rMS activates CAMKKβ is not clear. Transcranial magnetic stimulation and rTMS are currently recognized as indirect and noninvasive methods used to induce excitability changes by regulating intra- and extracellular ion concentrations, especially intracellular calcium concentrations (Grehl et al., 2015; Banerjee et al., 2017; Clarke et al., 2017). It seems that rTSMS activates CaMKKβ/AMPK–SOCS3 signaling activation depending on the focal rTSMS-induced increase in intracellular calcium levels. However, Jiang et al. (2022) showed that spinal microglia Ca^2+^ is related to their proliferation after pain. Therefore, rTSMS can activate AMPK by activating CaMKKβ, but the underlying mechanism is not well understood, which is one of the limitations of this study and the key focus of our future research.

### The focal repetitive trans-spinal magnetic stimulation parameters selected in this study

Another important factor to consider is the specific settings used for rTSMS treatment. Focal rTSMS parameters, particularly the frequency, intensity, and depth of magnetic stimulation, significantly influence the outcome of rTSMS therapy. Because rTSMS is a relatively new approach in NP research, the parameters used in this study were based on previous research with slight adjustments (Chalfouh et al., 2020). Our previous study showed that focal rTSMS at 20 Hz could promote the clearance of myelin debris by microglia to inhibit neuroinflammation in a SCI rat model (Zhai et al., 2024). Chalfouh et al. (2020) found that 10 Hz focal rTSMS improved lesion scars by decreasing fibrosis and inflammation in an SCI model. Another study demonstrated that initiating focal rTSMS at 15 Hz from 4 weeks after injury during the chronic stage attenuated the negative effects of SCI and stimulated functional recovery (Leydeker et al., 2013). A systematic review by Ferreira et al. (2024) investigated the effects of rTMS on non-neuronal cells in animal models of diseases. They found that most studies showed that high-frequency rTMS reduced the activity of astrocytes and microglia, leading to a decrease in the release of proinflammatory cytokines. On the basis of the above research, we selected high-frequency rTSMS for *in vivo* and *in vitro* studies.

Intensity is another important parameter. Several studies have proposed the motor threshold approach to determine the intensity of rTMS (Toledo et al., 2021b; Turi et al., 2021; Luo et al., 2022a). Luo et al. (2022b) showed that intensity higher than the rest motor threshold of rTMS has an anti-inflammatory effect. Our preliminary research (Zhai et al., 2024) and another study (Chalfouh et al., 2020) demonstrated that applying focal rTSMS with a specific strength (0.35 and 0.4 T, respectively) could reduce inflammation in the spinal cord. These approaches also resulted in tail twitch and improved function in animals with SCI. Considering that increasing stimulation intensity is also accompanied by increasing discomfort, to ensure comfort, tolerability, and effectiveness, the stimulation intensity in our study was set at 0.152 T (4% of the maximum power), which was the minimum stimulus intensity that caused involuntary twitch of both the hindlimb and tail in all rats.

Depth of stimulation is also very important. So far, we have not been able to determine the optimal depth of rTSMS. The purpose of the present study was to explore whether rTSMS has anti-inflammatory and analgesic effects, so whether the dorsal horn of the spinal cord was affected by the magnetic field was one of the key concerns of this study. To ensure that the dorsal horn of the spinal cord was affected by the magnetic field, we chose motor threshold as a depth biomarker of rTSMS. During magnetic stimulation, both hindlimbs and the tail showed involuntary contractions, suggesting that the ventral side of the spinal cord was affected, and the dorsal horn was also under the influence of the magnetic field at this time. In addition, Guadagnin et al. (2016) reported that the penetration depth of eight coils was 4 cm to the cortex, whereas circular coils are more prone to activate deeper brain structures. Therefore, the dorsal horn of the spinal cord was subject to magnetic stimulation. For rTSMS use in the clinic, we need to develop individualized treatment strategies. Prior to the formal treatment of the patient, we must determine the magnetic stimulation parameters by measuring motor-evoked potentials, in addition to analyzing the patient’s site of pain and determining the location of the corresponding segments by imaging examination.

This study has several limitations that should be acknowledged. First, although our *in vitro* experiments revealed that rMS activated CaMKKβ, we did not investigate the underlying mechanism. Several studies have demonstrated that neuronal or astrocytic Ca^2+^ increases in response to magnetic stimulation; however, changes in microglial Ca^2+^ after magnetic stimulation are still unknown. Whether rMS would change the intracellular Ca^2+^ concentrations in microglia, and whether the Ca^2+^ concentration is involved in mediating the microglial response to rMS, needs to be explored in the future. Second, we are the first group to focus on the effect of rTSMS on microglial SOCS3 in CCI-induced mechanical allodynia. In this study, we used SOCS3 siRNA to downregulate SOCS3 expression to verify whether the analgesic and anti-inflammatory effects of rTSMS are based on the upregulation of microglial SOCS3 in the spinal cord. However, the SOCS3 siRNA approach is not exclusive to these cells, and we did not specifically knockout/knockdown microglial SOCS3. We cannot claim that the effect of rTSMS is specific to microglia, which is the second limitation. However, the cells associated with inflammation in the spinal cord are mainly microglia cells and astrocytes, and we found that rTSMS could increase SOCS3 in microglia but not in astrocytes. Therefore, we suggest that the analgesic and anti-inflammatory effects of rTSMS are based on the upregulation of microglial SOCS3 in the spinal cord. Third, although our immunofluorescence results did not find that rTSMS increased neuronal SOCS3 levels, we cannot completely rule out that rTSMS does not up-regulate neuronal SOCS3. Therefore, we cannot exclude the possibility that rTSMS may also exert its beneficial effects on CCI-induced mechanical allodynia through SOCS3 expressed on neurons. This study focused on the anti-inflammatory effects of rTSMS, and microglia are the main cells involved in neuroinflammation. Therefore, microglia were the main target. Finally, we also found that astrocytes are activated in large quantities, and rTSMS can reverse phase activation. The present study aimed to explore whether rTSMS increases SOCS3 to induce anti-inflammatory and analgesic effects, and we found that rTSMS increased SOCS3 in microglia, rather than astrocytes, in the CCI model. Therefore, we did not discuss how rTSMS inhibits astrocyte activation.

In conclusion, this study proposes that noninvasive focal rTSMS has anti-inflammatory and analgesic effects on CCI-induced NP. *In vivo* and *in vitro* studies revealed that focal rTSMS can activate CaMKKβ/AMPK/SOCS3 signaling in microglia to inhibit neuroinflammation by inhibiting the p38/NF-κB and JAK/STAT3 signaling pathways. This study provides evidence that focal rTSMS induces therapeutic effects for NP in a preclinical rodent model and suggests possible translation to clinical application in humans.

## Additional files:

***Additional Figure 1:*** *Focal rTSMS does not affect the expression of SOCS3 in neurons in the dorsal horn.*

Additional Figure 1 Focal rTSMS does not affect the expression of SOCS3 in neurons in the dorsal horn.
(A, B) Representative immunofluorescence images showing that focal rTSMS did not affect the expression of SOCS3 in neurons in the dorsal horn. This dashed box shows the dorsal horn of the spinal cord. Scale bars: 100 μm. Data are expressed as mean ± SD (n = 3) and were analyzed by one-way analysis of variance followed by Tukey's multiple comparisons tests. CCI: Chronic constrictive injury; DAPI: 4',6-diamidino-2-phenylindole; rTSMS: repetitive trans-spinal magnetic stimulation; SOCS3: suppressor of cytokine signaling 3.

***Additional file 1:*** *Open peer review report 1.*

OPEN PEER REVIEW REPORT 1

## Data Availability

*All data relevant to the study are included in the article or uploaded as Additional files*.
