# Supplementary figures and images for: Trans-spinal magnetic stimulation upregulates microglial SOCS3 to attenuate neuroinflammation in chronic constriction injury–induced neuropathic pain
Source: Neural Regen Res. 2025 Apr 29;21(7):3092–102. doi: 10.4103/NRR.NRR-D-24-00912 (PMC13378957; doi:10.4103/NRR.NRR-D-24-00912)

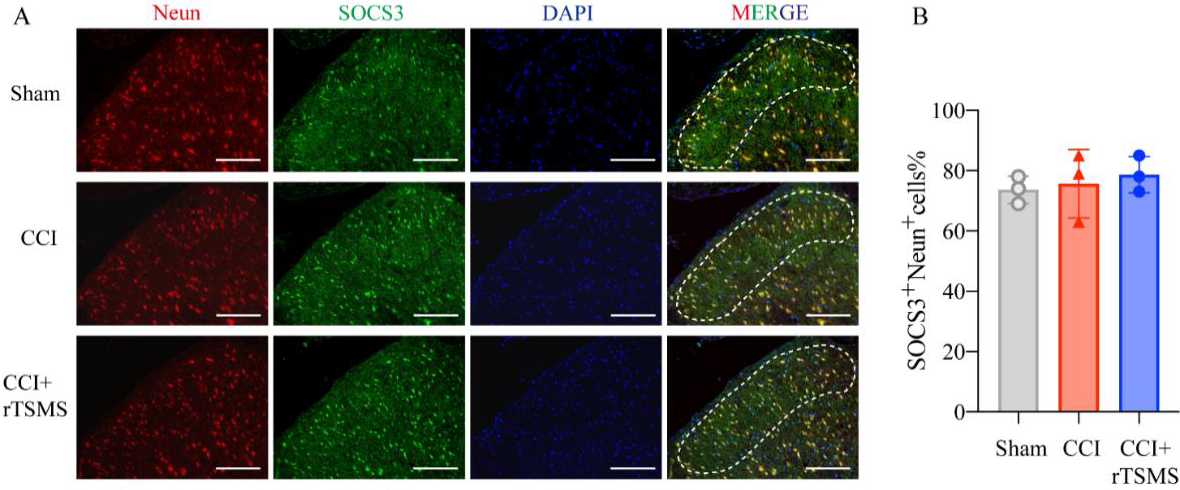

Supplement: Supplementary file 1 [file NRR-21-3092_Suppl1.tif]
