# Supplementary material for: Trans-spinal magnetic stimulation upregulates microglial SOCS3 to attenuate neuroinflammation in chronic constriction injury–induced neuropathic pain
Source: Neural Regen Res. 2025 Apr 29;21(7):3092–102. doi: 10.4103/NRR.NRR-D-24-00912 (PMC13378957; doi:10.4103/NRR.NRR-D-24-00912)
Supplement: Supplementary file 2 [file NRR-21-3092_Suppl1.pdf]

OPEN PEER REVIEW REPORT 1

Name of journal: Neural Regeneration Research
Manuscript NO: NRR-D-24-00912
Title: Trans-spinal Magnetic Stimulation upregulates microglial SOCS3 to attenuate neuroinflammation in CCI-induced neuropathic pain
Reviewer's Name: Nicolas Guerout
Reviewer's country: France

COMMENTS TO AUTHORS

The authors present a very interesting study on the effects of magnetic stimulation in a rat model of chronic pain. The results presented are very compelling. Additionally, the authors explore the signaling pathways that could explain these effects, particularly regarding the role that rTMS might play on microglial cells.
Nevertheless, I have a few comments to make to the authors.

Page 17, Figure 3 Results: The authors cannot claim that the effect is specific to microglia, as the siRNA approach is not exclusive to these cells.

Figure 1: Could the authors provide a lower magnification image of the spinal cord (SC) to show the entire SC? Additionally, could they perform immunohistochemistry (IHC) or Western blot (WB) to quantify the amount of substance P in the dorsal SC?

Figure 2: The authors should include a CD86 co-staining with Iba1.

Figure 3: The authors should calculate the ratio of $SOCS3^{+}$-$Iba1^{+}$ cells among the $Iba1^{+}$ population.

Figure 4: The authors should add a rMS without LPS group to assess the effects of rMS on non-activated cells.

Figure 6: The authors should perform c-Fos staining to evaluate the effects of compound C on rMS treatment.
